# When Nature Requires a Resource to Be Used—The Case of *Callinectes sapidus*: Distribution, Aggregation Patterns, and Spatial Structure in Northwest Europe, the Mediterranean Sea, and Adjacent Waters

**DOI:** 10.3390/biology13040279

**Published:** 2024-04-19

**Authors:** Luca Castriota, Manuela Falautano, Patrizia Perzia

**Affiliations:** Italian Institute for Environmental Protection and Research, Department for the Monitoring and Protection of the Environment and for the Conservation of Biodiversity, Unit for Conservation Management and Sustainable Use of Fish and Marine Resources, Lungomare Cristoforo Colombo 4521 (Ex Complesso Roosevelt), Località Addaura, 90149 Palermo, Italy; luca.castriota@isprambiente.it (L.C.); manuela.falautano@isprambiente.it (M.F.)

**Keywords:** alien species, biological invasions, Atlantic blue crab, ecological indicators, fishing gear, GIS, invasion dynamics, invasion hotspots, mitigation measures, spatio-temporal statistics

## Abstract

**Simple Summary:**

The Atlantic blue crab *Callinectes sapidus*, which is native to the western Atlantic coast and listed among the 100 most invasive alien species in the Mediterranean Sea, is attracting a great deal of interest because of its rapid colonisation of new areas, the significant increase in its population, and the impacts it may have on ecosystems. Outside its natural distribution range, the species was first found on the European coasts of the Atlantic in the early 1900s, and a few decades later, it was introduced into the Mediterranean Sea, probably through maritime traffic. Currently, it is found in almost the entire Mediterranean Basin and is also expanding into the Black Sea and along the north African and Iberian Atlantic coasts. This study describes the distribution of the Atlantic blue crab in Northwest Europe, in the Mediterranean Sea, and in adjacent waters through a series of ecological indicators elaborated using spatial–temporal statistics. The main results highlight that the species is expanding in the Mediterranean and adjacent waters, while in northern Europe, the population remains confined in some areas. The main species detection methods are analysed, finding that traps and nets are the most used methods, and management suggestions are provided.

**Abstract:**

The Atlantic blue crab *Callinectes sapidus*, which is native to the western Atlantic coast and listed among the 100 most invasive alien species in the Mediterranean Sea, is attracting a great deal of interest because of its rapid colonisation of new areas, the significant increase in its population, and the impacts it may have on ecosystems and ecosystem services. Outside its natural distribution range, the species was first found on European Atlantic coasts in the early 1900s and was introduced into the Mediterranean Sea a few decades later, probably through ballast water. Currently, it is found in almost the entire Mediterranean Basin and is also expanding into the Black Sea and along the north African and Iberian Atlantic coasts. Based on a systematic review of *C. sapidus* occurrences, this study describes its distribution, aggregation patterns, and spatial structure in Northwest Europe, the Mediterranean Sea, and adjacent waters through a series of ecological indicators elaborated using GIS spatial–temporal statistics. The main results highlight that the species is expanding in the Mediterranean and adjacent waters, while in northern Europe, the population remains confined in some areas. Furthermore, the main species detection methods are analysed, finding that traps and nets are the most frequently used methods, and management suggestions are provided.

## 1. Introduction

Rapid globalisation and increases in travel, trade, and transport have accelerated the introduction of alien species in recent decades. Some alien species that are introduced and successfully settled can rapidly expand, causing serious damage to native species and ecosystems often also accompanied by impacts on economic activities and effects on human health: these are invasive alien species (IASs) [1,2]. Invasive alien species represent one of the five most direct drivers of biodiversity loss, along with changes in land and sea use, the direct exploitation of species, climate change, and pollution. Around the world, more than 3500 species are invasive, with documented impacts of which 10% concern the marine environment [3]. Even though invasive alien species pose a serious global threat, they are still underestimated and often unacknowledged.

The Atlantic blue crab *Callinectes sapidus* Rathbun, 1896 (Brachyura: Portunidae) is a species native to the western Atlantic coast, ranging from Nova Scotia in Canada to northern Argentina, including Bermuda and the Antilles [4]. It has been listed among the 100 most invasive alien species in the Mediterranean Sea [5] and is attracting a great deal of interest because of its rapid colonisation of new areas, the significant increase in its population, and the impacts it may have on ecosystems and ecosystem services. It was recently observed in a western Mediterranean area that *C. sapidus* invasion has the potential to induce severe changes in the structure and composition of coastal marine and freshwater communities, with direct impacts on the protection of biodiversity and life in coastal human populations [6]. On the other hand, the species is a high-value resource in its native area and is also beginning to be appreciated in some Mediterranean fisheries [7,8].

In the early 1900s, *Callinectes sapidus* was found on the Atlantic coasts of Europe in 1900, while the first Mediterranean specimens were detected at the end of the 1940s [9], having probably been introduced via ballast water from transoceanic ships. The species has now spread to many areas of the basin, probably due to its high tolerance to environmental changes (e.g., temperature, salinity), high fecundity, strong swimming ability, and combative nature [4]. The Atlantic blue crab lives in coastal waters, estuaries, and lagoons on sandy and muddy bottoms with very high salinity and temperature variations. The life cycle of the species is very complex, with the use of both marine and estuarine habitats depending on the stage of development and sex. In particular, during the reproductive phase, after mating in brackish estuarine waters, females migrate to coastal waters with higher salinity to release their eggs and tend to remain there or move to nearby marine waters, while males prefer to remain in areas with low salinity [10,11]. After hatching, the larvae complete their development in coastal waters and enter brackish habitats in the post-larva stage until they reach the juvenile stage; both juveniles and adults can then be found in both freshwater and hyperhaline habitats [12,13,14]. However, patterns in the blue crab life cycle can vary by region or even habitat, while the timing and duration of life cycle events appear to vary with latitude [15,16].

The Atlantic blue crab is an opportunistic and necrophagous dominant predator capable of regulating benthic prey populations. Its broad dietary spectrum includes plants, detritus, polychaetes, molluscs, crustaceans, and fish [15,17,18,19]; the last three categories are its main prey [20]. The species also shows a great ability to vary its energy sources in relation to seasons, environmental conditions, and life cycle; this adaptability could represent a key determining factor in the success of its invasion [21].

To date, *Callinectes sapidus* has colonised much of the Mediterranean Basin and adjacent waters as well as Northwest Europe. In some areas, it has shown an invasive character [6,8,22] so considerable as to cause a negative socio-economic impact [22] and to require urgent action to contain the population. Mapping and monitoring the distribution of an invasive species is essential to understanding its spread and thus identifying areas of further expansion [23,24,25] in which to intervene with management actions [24]. From this perspective, occurrences of *C. sapidus* in the Mediterranean Sea and adjacent waters as well as in Northwest Europe are analysed in order to provide an overview of its spatial distribution and to identify the directional trends of its spread. An analysis of methods of capturing and detecting the Atlantic blue crab was also carried out in order to identify the main equipment used and its distribution for management purposes.

## 2. Materials and Methods

### 2.1. Callinectes sapidus Invasion History

All the available data and information on *Callinectes sapidus* in Northwest Europe (NWE) and the Mediterranean and adjacent waters including the eastern Atlantic Ocean and Black Sea (MAW), were collected and reviewed according to the Preferred Reporting Items for Systematic Reviews and Meta-Analyses (PRISMA) [26].

Relevant articles in the literature were identified through the following combinations of keywords: “*Callinectes sapidus*”, “blue crab”, “Atlantic blue crab”, “*Callinectes diacanthus*” (an old synonym of *C. sapidus*), “Mediterranean Sea”, “Black Sea”, “Northwest Europe”, and “European coastal waters”. Freely accessible web search engines for academic articles, i.e., Google Scholar (scholar.google.com) and ResearchGate (researchgate.net), were used (accessed on 15 January 2024); the main scientific journals that address bioinvasion topics were also consulted. Other bibliographic sources were obtained from the references listed in the articles found.

Data from studies were extracted and organised into a geodatabase including the year of occurrence (if missing, the publication year was used), location, country, sea, geographic coordinates, method of detection (capture/observation), and references. According to Castriota et al. [24], geographic coordinates were assigned a position accuracy value: 1—exact coordinates as reported in the bibliographic source or taken from a detailed map; 2—coordinates derived from a specific sighting/capture site, e.g., a city name; 3—coordinates derived from a generic sighting/capture site, e.g., a gulf name; and 4—coordinates derived from a highly generic location, e.g., a sea name.

Duplicate information as well as doubtful records, e.g., cases of misidentification or indications of the common name “blue crab” only, were excluded. The studies from the review can be found in the Supplementary Materials (Table S1).

### 2.2. Distribution, Aggregation Pattern, and Spatial Structure Analyses

In order to reduce the effect of possible preferential sampling, which can lead to an error in distribution modelling [27], only the first record within a 0.05° Lat/Long grid was considered for all spatial analyses, disregarding the number of specimens [24].

The data were analysed separately for the two areas investigated, i.e., NWE and the MAW, based on temporal and geographical differences in the invasion.

According to Perzia et al. [25], a series of statistical analyses were carried out, and ecological indicators were examined in order to study (i) temporal and spatial–temporal patterns; (ii) aggregation patterns and spatial structure; and (iii) the key characteristics of distribution. The same set of analyses and indicators was applied for both areas (NWE and the MAW) is reported in Table 1.

The cumulative curves of *Callinectes sapidus* occurrences in NWE and the MAW were calculated in order to identify the occurrence increase and the invasion phases, as well as the expansion areas in terms of the cumulative number of 0.05° Lat/Long cells affected by the occurrences over time. The invasion phases were identified based on the most evident changes in slope of the cumulative curves; for each interval, the slope of the regression line obtained using the least squares method was calculated to obtain the different rates of increase in the number of new occurrences over time [25]. The expansion increase is indicated by the *y*-axis values of the cumulative curves.

Quantitative multi-parameter modelling of the dataset was performed using the “Density” toolset in the ArcGIS spatial analysis toolbox and “Analysing patterns”, “Mapping clusters” and “Measuring geographic distributions” toolsets in the ArcGIS spatial statistics toolbox in order to describe the distribution, aggregation patterns, and spatial structure of *Callinectes sapidus* occurrences in the study areas [28].

Cumulative kernel density maps were elaborated for different periods to evaluate, from a qualitative perspective, occurrences of density increases over time and space (species expansion areas) and to identify persistent areas of occurrence (high-density areas) of *C. sapidus*.

The aggregation patterns and spatial structures of the records were studied in order to find (i) the spatial pattern of distribution, (ii) the change in the distribution pattern over time, (iii) the earliest and latest instances of species spread, (iv) dispersion and/or settled areas, and (v) outliers. The aggregation patterns were analysed using the global Moran’s index of spatial autocorrelation (GMI) to evaluate if occurrences were clustered, dispersed, or random. The index value is between −1 (dispersion) and 1 (clustering) [29].

The spatial structures of the two areas were analysed using an optimised hot spot analysis (O–GOG*). This analysis uses the Getis–Ord Gi* statistic and evaluates the characteristics of the input feature class, identifying an appropriate scale of analysis to produce optimal results [29]. Given occurrence points and years, an O–GOG* analysis creates a map of statistically hot (more recent years) and cold (initial years) spots and a density surface raster layer using the kernel density tool [30]. The Anselin local Moran’s I analysis (AMI) was also used out to identify outlier records (i.e., recent records in proximity to a group of older records and vice versa).

The key characteristics of the distributions—i.e., the centre of gravity (mean centre–median centre), directional dispersion (XStdDist, YStdDist), and directional trends were measured to track distribution changes over time and space and to compare the time groups of occurrences with each other.

### 2.3. Callinectes sapidus Detection Methods

Methods of capturing/observing *Callinectes sapidus* reported in the bibliographic sources were analysed in terms of the percent frequency of occurrence. Fishing gear was grouped into categories in the first tier, according to Lucchetti et al. [31], as follows: dredges including dredges, scallop dredges, and hydraulic dredges; falling gears including throw nets and howk nets; gillnets and entangling nets; hooks and lines including rods/line, angling, hooks, and pins; lift nets; miscellaneous gear including spearguns, harpoon, electric fishing equipment, and hand implements; seine nets; traps including basket traps, stationary nets, barrier traps, fyke nets, star traps, pots, eel cages, funnel traps, hoop traps, and wire traps; and trawls including beam trawls, bottom trawls, and trawls. A further category of fishing gear, i.e., unspecified nets, was created to include generic nets. Two more detection categories not attributable to fishing gear, i.e., visual observations and scientific sampling devices, were included. The distribution of the most represented categories of detection methods in the two areas of invasion was mapped.

## 3. Results

The literature search resulted in 4875 studies: 3645 academic articles from web search engines and 1230 from scientific journals on bioinvasion (the reference identification step). After the screening step (the removal of duplicates and non-informative studies), the remaining 268 studies, published from 1901 to 2023, were assessed for eligibility and considered for review. They included 1870 records of *Callinectes sapidus*, 60 in Northwest Europe and 1810 in the Mediterranean Sea and adjacent waters.

Regarding geographic position accuracy, there were 1347 records with a value equal to 1, 456 with a value equal to 2, 652 with a value equal to 3, and 2 with a value equal to 4. The full database used for data processing is reported in the Supplementary Materials (Table S1).

The total number of occurrences selected for the analyses was 915: 869 in the MAW and 46 in the NWE area.

### 3.1. Invasion History and Spatial–Temporal Patterns of Callinectes sapidus Distribution

**Figure 1:**
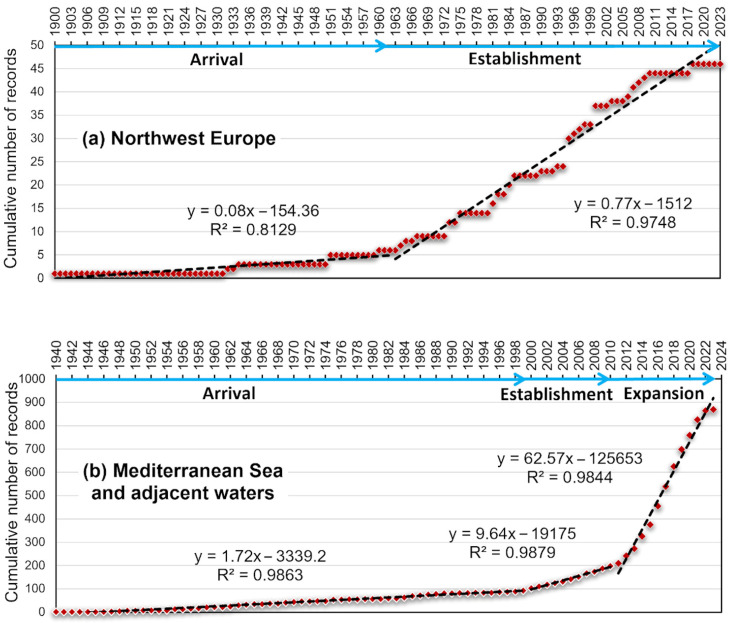
Cumulative curves of occurrences of *Callinectes sapidus* (red dotted lines) in the two identified invasion areas, with indications of the phases in the invasion process: arrival, establishment, and expansion. Equations of the regression lines (black dotted lines) with corresponding R^2^ values are also reported for (**a**) Northwest Europe and (**b**) the Mediterranean Sea and adjacent waters (the eastern Atlantic Ocean and the Black Sea). Only the first records within a 0.05° Lat/Long grid were considered for analysis. The cumulative number of records also corresponds to the cumulative number of cells affected by the occurrences over time. Blue arrows indicate invasion phases.

Figure 1a,b report the cumulative curves of *Callinectes sapidus* occurrences in the two invasion areas, NWE and the MAW, respectively, as well as the cumulative number of cells affected by the presence of the species over time.

The cumulative curve resulting from occurrences in the NWE area showed two slope changes corresponding to arrival phases from 1900 to 1962 (slope = 0.08 ± 0.005) and establishment from 1963 to 2023 (slope = 0.77 ± 0.02) in the invasion process; no expansion phase was detected in that area. The equations of the regression lines with corresponding R^2^ values are reported in Figure 1.

In the MAW area, the cumulative curve showed three evident slope changes (Figure 1), indicated here as (i) arrival, from 1940 to 1999; (ii) establishment, from 2000 to 2010; and (iii) expansion, from 2011 to 2023.

The positive slope variations indicated that the *Callinectes sapidus* invasion in the MAW area has grown much faster in the last twelve years (with a slope of 62.57 ± 2.37; the expansion phase) than during the previous seventy years (the arrival and establishment phases, with slopes of 0.91 ± 0.02 and 1.72 ± 0.03, respectively).

**Figure 2:**
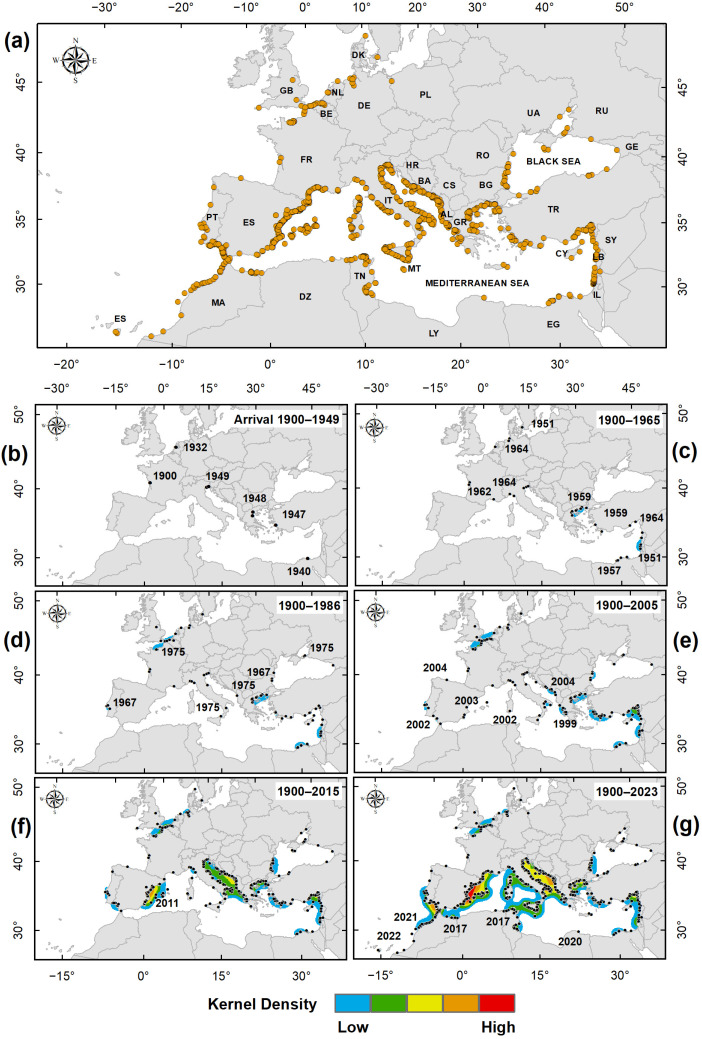
Distribution of *Callinectes sapidus* in Northwest Europe and in the Mediterranean Sea and adjacent waters together with kernel density cumulative maps. (**a**) The overall distribution of the selected records (the first record within a 0.05° Lat/Long grid); (**b**–**g**) Period-to-period variations in space and time of occurrence, corresponding to presumed invasion phases. The yellow and black circles in (**a**–**g**) indicate the records of *C. sapidus.* ISO 3166 Country Codes were used to indicate the countries in which *C. sapidus* occurs.

The overall distribution of records of *C. sapidus* in NWE and the MAW area is reported in Figure 2a, together with density cumulative maps (Figure 2b–g). These show period-to-period variations in space and time, highlighting substantial changes in occurrences in the two areas in terms of both spread and species aggregation.

In the NWE area, the species was found on the Atlantic coast of France in 1900 and spread in the Netherlands, Germany, and Denmark, with isolated occurrences until 1965 (Figure 2b,c). The first nucleus of aggregation appeared from 1975, and the density value was low (Figure 2d). The aggregation area expanded over time, but the density value remained almost constant until 2018, the year of the last occurrence in that area (Figure 2e–g).

In the MAW area, the first occurrence of *Callinectes sapidus* was along the Egyptian coast in 1940; the next occurrences were in the Aegean Sea in 1947 and 1948 and in the north Adriatic Sea in 1949 (Figure 2b). The first nuclei of aggregation appeared in 1951 in the Levantine Basin and in the Aegean Sea in 1959 (Figure 2c,d). In 1967, the first records of its occurrence in the Black Sea appeared. These nuclei were strengthened over time by other occurrences in the immediate neighbouring areas, and other nuclei appeared subsequently along the Mediterranean Turkish coast in the southern Adriatic and in the Ionian Sea; in the same period, occurrences in the western Mediterranean Sea were still sporadic and dispersed (Figure 2e). Since 2006, *C. sapidus* has spread rapidly in the western Mediterranean Basin, where areas of medium-density occurrences are very clear in the Adriatic Sea (green and yellow areas in Figure 2f) and along the east coast of Spain (yellow and orange areas). In the last eight years, 2016–2023, the density of this nucleus assumed particular importance, intensifying to high values, another nucleus appeared along the Atlantic coasts of Spain and Morocco, reaching the Canary Islands in 2022 (Figure 2g).

### 3.2. Aggregation Patterns and Spatial Structure

The distribution of *Callinectes sapidus* in NWE shows a weak spatial autocorrelation at the global scale (GMI expected index = − 0.022; GMI = 0.23; z > 2.58; *p* < 0.01), indicating a change in the spatial pattern over time. A similar pattern of aggregation was highlighted in the MAW (GMI expected index = − 0.001; GMI = 0.21; z > 2.58; *p* < 0.01).

**Figure 3:**
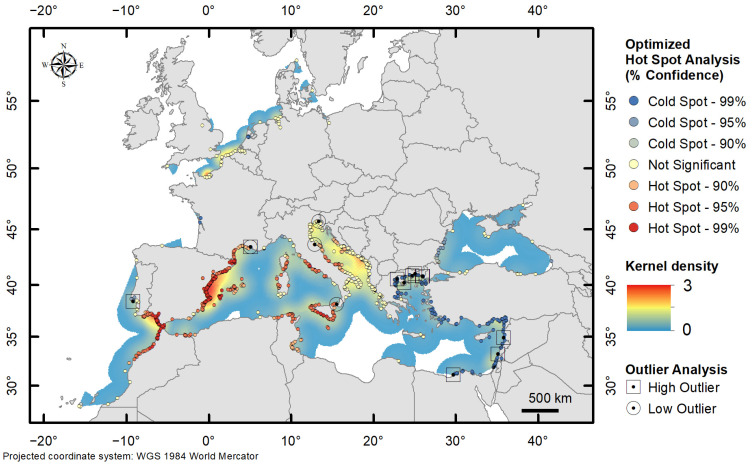
The results of the optimised hot spot analysis and outlier analysis on records of *Callinectes sapidus* in Northwest Europe and in the Mediterranean Sea and adjacent waters. Areas with statistically significant spatial clustering (hot spots = circles in red shades and cold spots = circles in blue shades), high outliers (uncoloured squares), and low outliers (uncoloured circles) were detected. Yellow circles indicate records with non-significant index values.

Figure 3 provides an overview of the optimised hot spot analyses (with corresponding kernel density values) and the outliers:

- NEW—at a local spatial scale, the O–GOG* analysis showed two areas with statistically significant cold spots (99%) along the Atlantic coast of France and the Netherlands (corresponding to older records of *Callinectes sapidus*). The other records had non-significant index values. No hot spots and spatial outliers were detected. Medium density values were recorded in the NWE area.
- MAW—the O–GOG* analysis showed a very statistically significant cold spot (99%) in the Levantine Basin corresponding to the initial direction of spread. Hot spots (99%) were detected in the western Mediterranean Basin and eastern Atlantic Ocean. Maximum density values were recorded in the same area. Hot spots of up to 95% were also detected along the Tyrrhenian Coast of Italy and around Sicily. The occurrences in the Adriatic and Ionian Sea had non-significant index values. High spatial outliers were detected in the Levant and north Aegean Sea, and low outliers were detected in the central Mediterranean Sea.

### 3.3. Key Characteristics of Distribution

**Figure 4:**
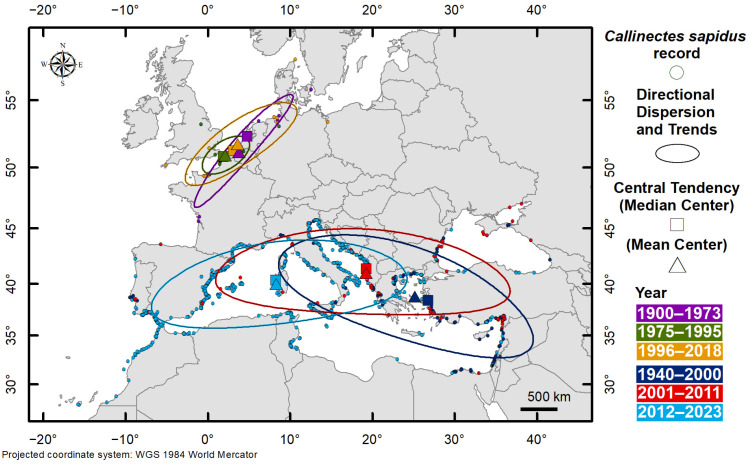
Key distribution characteristics of *Callinectes sapidus* in Northwest Europe and in the Mediterranean Sea and adjacent waters. The central tendency (measured as mean and median centres), directional dispersion, and trends, calculated for the two areas in different periods, show distribution changes in space and time.

Table 2 and Figure 4 provide the key characteristics of *Callinectes sapidus*’s distribution in NWE and the MAW, calculated per period.

The central tendencies (mean and median centres), the directional dispersions, and directional trends changed in space and time in the two areas:

- NWE—from 1900, i.e., the year of the first record on the Atlantic coasts of France, to 1973, the central tendencies, measured as median and mean centres, were found in the Netherlands and Belgium, respectively. The directional dispersion of distribution and trends was concentrated along the coast, from the southwest (France) to the northeast (Denmark), with a very elongated and narrow ellipse. In the second period, i.e., 1975–1995, there is an evident contraction of the ellipse, with a strong reduction in y dispersion (from 631 to 231 km) and median and mean centres approaching each other. In the 1996–2018 period, the central tendencies diverge somewhat, and the distribution is dispersed again along the *x*-axis with the maximum XStdDist recorded, changing its shape but not its direction.
- MAW—from 1940, i.e., the year of the first record on the Egyptian coast, to 2000, the central tendencies, measured as median and mean centres, were found in the Aegean Sea, close to each other. The directional dispersion of distribution and trends was from southeast, in the Levantine Basin, to northwest, in the central Mediterranean Sea. In the 2001–2011 period, the central tendencies were in the southern Adriatic (Albanian coasts). The distribution’s directional dispersion changed in shape and direction, showing a considerable westward dispersion: the XStdDist ranged from 1459 km to 437 and the YStdDist from 494 km to 1552 (Table 2). This period showed the highest dispersion of *Callinectes sapidus* over time. This expansion is also confirmed by the distribution key characteristics in the third period (2012–2023). The ellipse is elongated from Greece to Spain, and the direction extends towards the Strait of Gibraltar. Central tendencies are shifted further west, on the western coast of Sardinia.

### 3.4. Callinectes sapidus Detection Methods

Out of a total of 1878 records, 1346 contained information about the method of capture/observation. Methods of detecting *Callinectes sapidus* in the investigated areas included the use of fishing gear (681 records), sampling devices (14 records), and visual detection (126 records).

**Figure 5:**
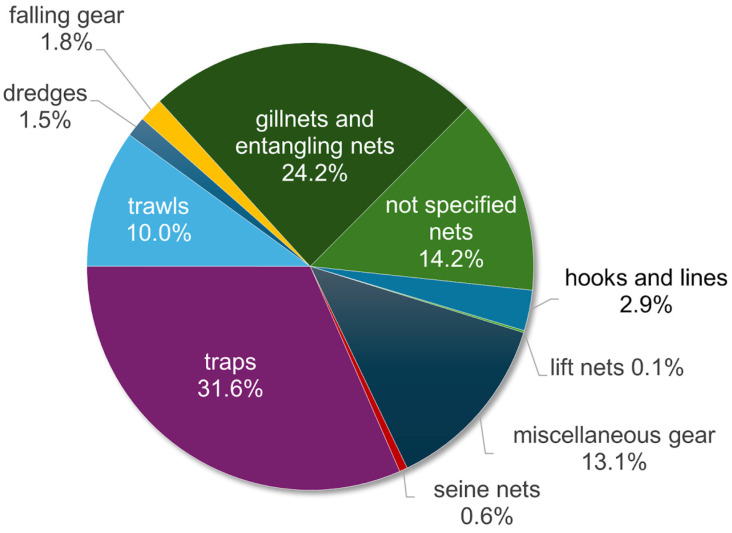
The frequency of occurrence of *Callinectes sapidus* fishing gear categories from an analysis of the literature.

Taking into account fishing gear only, the most represented category used for catching *Callinectes sapidus* specimens was traps, accounting for 32% of the identified gear categories, followed by gillnets and entangling nets (24%) and unspecified nets (14%) (Figure 5). Miscellaneous gear accounted for 13%, trawls for 10%, and hooks and lines for 3%.

**Figure 6:**
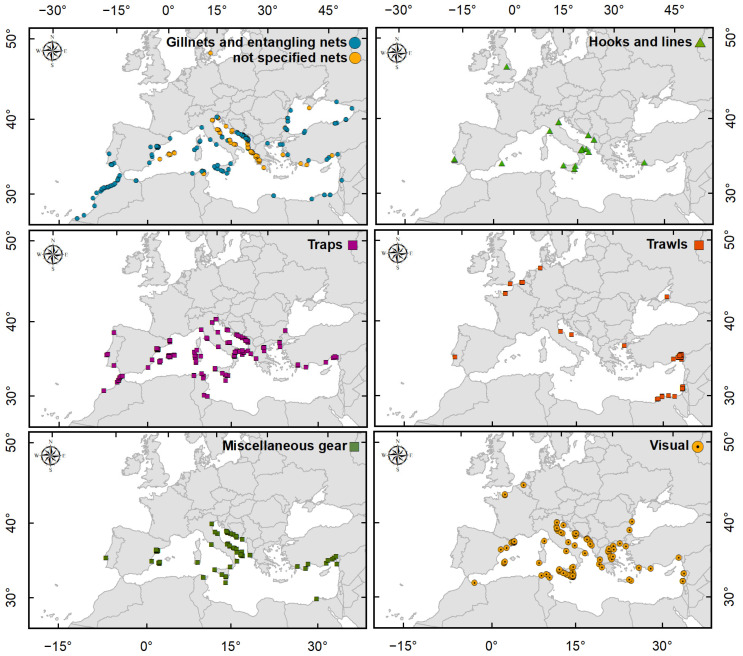
Distribution maps of the most-represented categories of *Callinectes sapidus* detection methods.

The distribution of the most-represented gear categories (Figure 6) showed that nets (gillnets and entangling nets plus unspecified nets) are distributed almost throughout the MAW area. The traps category was also well distributed in the MAW area except for the Black Sea and southeastern Mediterranean. The trawls category was mainly distributed in the NWE as well as in the Levantine Basin. Hooks and lines were mainly used in Italy. Miscellaneous gear was distributed, with numerous records of its use in the Adriatic Sea. Finally visual observations were mapped throughout almost the entire Mediterranean Basin, with high concentrations in Greece and Italy.

## 4. Discussion

The first signs of the invasion of European waters by *Callinectes sapidus* began in 1900, when an individual was found on the French Atlantic coasts and, after about thirty years, the species was reported in the Netherlands, Denmark, and Germany [9]. The invasion process was very slow, and there were no significant environmental impacts reported in those areas at that time. Further sightings were later recovered in these areas, with more or less confirmed signs of expansion eastward into Poland and westward into the United Kingdom, as also shown by the directional dispersion change in the last period considered, 1996–2018, compared to the previous ones. Also, the kernel density maps show very slight changes in invasion after 1965, with no significant variation until the present times. The analysis of the cumulative number of cells (expansion areas) in this area shows a plateau phase of about 60 years, i.e., a lag time corresponding to the arrival phase, followed by a slow, discontinuous increase in records, indicating that the species is still in an early stage of establishment. This consideration is further reinforced by the O–GOG* analysis that identified only cold spots, indicating an absence of recent significant expansion. Also, the key characteristics of the distribution indicate that the species remains confined in about the same area. A possible explanation for the slow colonisation of northern Europe by this species could reside in its low water temperatures. According to laboratory experiments [32], Atlantic blue crab larvae would require water warmer than 21 °C for their development, temperature conditions not very frequent in the northern seas. The importance of temperature, as a key environmental factor for the successful establishment of the Atlantic blue crab, is also corroborated by a recent study on its thermal tolerance based on the species’ metabolic response to a wide temperature range, showing that the thermal optimum for the species is around 24 °C [33].

The invasion of *Callinectes sapidus* in the MAW area began almost simultaneously in the 1940s from several outbreaks scattered in different Mediterranean areas. According to the literature, the first documented findings took place in the upper Adriatic [34], in two distinct parts of the Aegean [35], in Egyptian coastal lakes [36] (Figure 2b), and later in the northwestern Mediterranean [37,38] (Figure 2c). However, while increases in records of occurrences in the Aegean and Levantine Basin are observed (Figure 2c), the Adriatic suffered the invasion of the Atlantic blue crab much later (Figure 2d and the following figures), suggesting that the first Adriatic records were accidental and isolated events. The successful colonisation of the Adriatic could therefore have happened by unaided secondary dispersion, probably starting from the Albanian coastal lakes (Figure 2e) where a *C. sapidus* population had already settled by the early 2000s [39].

Almost at the same time, in the 2006–2015 period (Figure 2f), there was a greater presence of Atlantic blue crab records in the western Black Sea, possibly related to global warming, particularly the water temperature increase experienced by this area between 1979 and 2018 [40]. After this period of increase, there are no substantial increases in the population (Figure 2f,g), which may still be in its arrival phase, and the establishment phase may manifest itself in the future.

The optimised hot spot identified parts of the Aegean Sea and Levantine Basin as significant cold spots, indicating these areas as promoters of the process of the invasion of the Atlantic blue crab in the Mediterranean. On the contrary, in the western Mediterranean Basin and the East Central Atlantic, significant hotspots were found, indicating recent, strong colonisation by the species. Such a trend is also highlighted by the strong east–west shift of the central tendencies over the three periods considered. The kernel density maps showed period-to-period variations in space and time, highlighting the substantial changes in *Callinectes sapidus* occurrences in the Mediterranean Sea. In particular, the most significant changes occurred after 2005 (Figure 2f), when some invasion areas began to intensify, i.e., the Adriatic Sea and the western Mediterranean Basin, and even more after 2015 (Figure 2g), which included the Atlantic coasts of North Africa, the Canary Islands, and the Iberian Peninsula. In the Adriatic and Ionian Seas, the species is clearly established, according to the insignificant O–GOG* values.

The cumulative record number analysis in this area identified three phases of the invasion: the arrival phase lasted about 60 years, the establishment phase lasted about 10 years, and the expansion phase is still ongoing.

The analysis of the methods of detecting *Callinectes sapidus* revealed that most of the records reported in the literature are attributable to catches by fishing gear and, among these, that traps and nets (gillnets and entangling nets plus unspecified nets) are the most represented in the number of records and the most extensively distributed types of fishing gear in the MAW area. These are small-scale fishing gears used in shallow coastal waters, lagoons, and estuaries. The use of trawls is limited compared to other gear and is concentrated principally in NWE, as well as in Egypt, Israel, and Turkey where the species was caught on soft, muddy bottoms. In reference to the miscellaneous gear category, several records are attributable to recreational fishing, mainly using spearguns. Lastly, the numerous visual records mapped in the Mediterranean area, with high concentrations in the Adriatic and Aegean Seas, Sicily, and Spain, are related to great research effort, also including participative methods of data collection [41,42,43,44,45]. These results suggest that artisanal fishers and citizens are the best actors to be involved in surveillance and early detection activities aimed at detecting *C. sapidus*, as was successfully experienced in the case of the invasion of the blue swimming crab *Portunus segnis* in the Pelagie Islands [24,46].

### Callinectes sapidus in the Mediterranean Sea and Adjacent Waters: A Resource to Be Used

Concern over the danger of the Atlantic blue crab invasion had already been highlighted at the beginning of its discovery along Greek coasts in the 1950s, when its impact on other native crabs and lagoon fishing activities was denounced and when intensive fishing and canning were identified as the only defences against its invasion [35].

Given the extent of the invasion, it is unthinkable to try to eradicate the Atlantic blue crab from the invaded habitats. However, containment and control actions can be implemented to reduce the population, at least on a local scale. As a result of numerous reports from citizens, various awareness-raising campaigns were launched in 2023 from several fronts inviting citizens to consume the Atlantic blue crab [47,48,49]. Incentivising the consumption of IAS would prove to be a viable option to both limit the proliferation of problematic species and to provide a source of fresh local food [50]. This apparently applicable suggestion, however, leaves some doubts regarding the health aspect of the resource: *Callinectes sapidus* individuals do, in fact, live in both fresh [51] and brackish water and sea water and could therefore be subject to uncontrolled contamination by pollutants, particularly in estuaries and river mouths where industrial and domestic discharges are very likely to converge and where the species abounds. The accumulation of contaminants and microplastics in Atlantic blue crab tissues and the risks of their consumption by humans are reported in several studies from different parts of the world [52,53,54,55,56,57], although contamination levels vary depending on the location. This is why it is preferable not to give instructions in this regard to the many citizens who ask what to do in case of sighting or catching an Atlantic blue crab. However, the idea of blue crab consumption as a strategy to contain the population, as proposed by Mancinelli et al. [58], could be realised more efficiently if the crab was officially included in fish markets as a safe and health-controlled fishing resource. Such a solution was considered by Italy among the programs of measures under the Marine Strategy Framework Directive (2008/56/CE), also including other edible invasive alien species, with the aim of tracing their catches and sales, thus also obtaining reliable data on population abundance. This action must, however, be accompanied by awareness-raising campaigns to promote the product and its marketing and to adopt environmentally friendly behaviours and choices. Until now, in fact, the species informally required by the local fish markets, particularly those of the northern Adriatic, concerned only adult males as they are larger, and therefore of greater economic value, compared to females and juveniles. This market demand could have led fishermen to discard small-sized juvenile individuals as well as pregnant females carrying millions of eggs into the environment, thus favouring the success of the population. After having suffered the proliferation of the Atlantic blue crab in north Adriatic waters, which particularly affected shellfish farmers, *C. sapidus* was officially included on the Italian list of species of commercial interest by a ministerial decree [59], and its capture and disposal were encouraged by national tax incentives [60]. This could be the beginning of a wider chain that exploits the Atlantic blue crab not only for food purposes but also for the food industry and for the extraction of natural polymers like chitin and chitosan, which are used in biomedical engineering [61,62]. Such a chain would also intercept small-sized, non-marketable individuals for alimentary purposes, therefore avoiding their reintroduction in the environment with known consequences.

Another important aspect to consider is the fishing gear to be adopted from an adaptive management perspective. Since the Atlantic blue crab lives part of its life cycle in sensitive environments in which the use of invasive extraction methods, such as trawling, would have a high impact on the environment, it is strongly advised to adopt a selective, almost monospecific, fishery like the trotline, a short longline that can be used near shore, along streams, or in shallow water bodies. In the Chesapeake Bay region, Maryland, where *Callinectes sapidus* is native and provides a fishery resource of high commercial value, the trotline has over the years one of the dominant types of gear [63]. The use of trotlines or other lines could also be encouraged through the involvement of experienced fishermen for the purpose of crab population containment. Such gear, being less invasive than others, can be very suitable in sensitive environments, e.g., protected areas, lagoons, or sites with or fragile habitats. As an alternative to lines, traps can be adopted for population containment, also the wide use recorded in the studied area. Both are passive types of gear, as environmentally friendly as they are selective and not disruptive, and they permit the release of non-target organisms almost undamaged; in addition, they are economical and easy to use. Fishing with selective gear in combination with the application of models able to estimate the density/abundance of the invasive population [64] could also prove useful, especially in confined environments, for converting this harmful invasive species into a sustainable seafood product through targeted population control.

## 5. Conclusions

This paper provides an update on the distribution of *Callinectes sapidus* in Northwest Europe and in the Mediterranean Sea and adjacent waters. For the first time, spatial–temporal statistics were applied to identify its invasion phases, pathways, and directional trends in spread and main settlement areas. The analysis carried out on the main methods of species detection and their distribution in the invasion areas highlighted the importance of passive gear in the catching the Atlantic blue crab and the role of citizens in reporting it, especially in areas not frequented by fishers.

The management of the Atlantic blue crab is crucial for safeguarding both the environment and related ecosystem services. The high availability of the crab, its high commercial value, and the exploding demand by consumers has favoured the birth of startups also dedicated to its overseas export for food purposes. However, such a virtuous response cannot be the only solution since in some areas, the crab population does not reach the quantities required to export it but has enough of a presence to impact the environment and local economy. Given the scale of the invasion in recent years, local governments need urgent responses regarding the management of the phenomenon from decision-makers. Surely the first fundamental step to preventing and containing a bioinvasion is the maintenance of the integrity of the marine environment. In fact, a healthy environment and its associated communities can better counteract the expansion of a new species. Any management action must, in any case, take into account the conservation of the environment in order not to add an additional stress beyond that determined by the presence of the invasive species.

## Figures and Tables

**Table 1:** Analyses of spatial and temporal indicators and their ecological meaning, including methods and spatial and time scales used (modified from Perzia et al. [25]). Only the first records within a 0.05° Lat/Long grid were considered for analysis. NWE—Northwest Europe area; MAW—Mediterranean and adjacent waters area.

Analysis/Indicator Name	Tools	Spatial Scale	Time Scale	Ecological Meaning
**Temporal and spatial–temporal pattern**				
Occurrence increase	Cumulative curve of occurrence	Global	All years	Occurrences increasing over time and space. Identification of invasion phases
Occurrence increase rate	Evaluation of the slopes of the cumulative curve by the Least Squares Method	Global	NWE: 1900–1962 1963–2018 MAW: 1940–1999 2000–2010 2011–2023	The rate of specimens increasing over time
Density hotspots	Kernel density	Global	1900–1949 1900–1965 1900–1986 1900–2005 1900–2015 1900–2023	Expansion areas; nuclei of record aggregation; persistent occurrence areas; space–time occurrence density increase; highest-density areas
**Aggregation patterns and spatial structure**				
Global spatial autocorrelation	Global Moran’s I (GMI) NWE: cutoff distance = 350 km MAW: cutoff distance = 350 km	Global	All years	Distribution pattern: dispersion vs. random vs. clustering; change in spatial pattern over time
Statistically significant hot spots and cold spots	Optimised hot spot analysis (O–GOG*) NWE: distance band = 95 km MAW: distance band = 200 km	Local	All years	Initial and current direction of spread and identification of dispersion/settle areas
Spatial outliers	Anselin local Moran’s I (AMI) Cluster and outlier analysis NWE: distance band = 95 km MAW: distance band = 200 km	Local	All years	Recent records in proximity of group of older records and vice versa
**Key characteristics of distribution**				
Centre of gravity	Central tendency (mean centre—median centre)	Global	NWE: 1900–1973 1975–1995 1996–2018 MAW: 1940–2000 2001–2011 2012–2023	Species concentration centre and its change over time
Directional dispersion	XStdDist and YStdDist (km); standard deviational ellipse (1 standard deviation)	Global		Species distribution in X and Y directions
Directional trends	Rotation (°) Standard deviational ellipse (1 standard deviation)	Global		Directional trend in species dispersion

**Table 2:** Values of the directional dispersion and directional trends of *Callinectes sapidus* record distributions in Northwest Europe (NWE) and in the Mediterranean Sea and adjacent waters (MAW), calculated per period.

Area	Years	Directional Dispersion XStdDist (km)	Directional Dispersion YStdDist (km)	Directional Trend Rotation (°)
NWE	1900–1973	97	631	45
	1975–1995	119	231	61
	1996–2018	175	558	59
MAW	1940–2000	1459	494	99
	2001–2011	438	1553	86
	2012–2023	425	1356	83

## Data Availability

The original contributions presented in the study are included in the article and Supplementary Materials, further inquiries can be directed to the corresponding author.

## Supplementary Materials

The following supporting information can be downloaded at: https://www.mdpi.com/article/10.3390/biology13040279/s1, Table S1: *Callinectes sapidus* occurrences database (References [65,66,67,68,69,70,71,72,73,74,75,76,77,78,79,80,81,82,83,84,85,86,87,88,89,90,91,92,93,94,95,96,97,98,99,100,101,102,103,104,105,106,107,108,109,110,111,112,113,114,115,116,117,118,119,120,121,122,123,124,125,126,127,128,129,130,131,132,133,134,135,136,137,138,139,140,141,142,143,144,145,146,147,148,149,150,151,152,153,154,155,156,157,158,159,160,161,162,163,164,165,166,167,168,169,170,171,172,173,174,175,176,177,178,179,180,181,182,183,184,185,186,187,188,189,190,191,192,193,194,195,196,197,198,199,200,201,202,203,204,205,206,207,208,209,210,211,212,213,214,215,216,217,218,219,220,221,222,223,224,225,226,227,228,229,230,231,232,233,234,235,236,237,238,239,240,241,242,243,244,245,246,247,248,249,250,251,252,253,254,255,256,257,258,259,260,261,262,263,264,265,266,267,268,269,270,271,272,273,274,275,276,277,278,279,280,281,282,283,284,285,286,287,288,289,290,291,292,293,294,295,296,297,298,299,300,301,302,303,304] are cited in the Supplementary Materials).
